# Antimicrobial and Antibiofilm Effects of Human Amniotic/Chorionic Membrane Extract on *Streptococcus pneumoniae*

**DOI:** 10.3389/fmicb.2017.01948

**Published:** 2017-10-10

**Authors:** Mukesh K. Yadav, Yoon Y. Go, Shin Hye Kim, Sung-Won Chae, Jae-Jun Song

**Affiliations:** ^1^Department of Otorhinolaryngology-Head and Neck Surgery, Korea University College of Medicine, Seoul, South Korea; ^2^Institute for Medical Device Clinical Trials, Korea University College of Medicine, Seoul, South Korea

**Keywords:** *Streptococcus pneumoniae*, biofilm, amniotic-membrane, chorionic-membrane, antimicrobial, antibiofilm, proteomics

## Abstract

**Background:** *Streptococcus pneumoniae* colonize the human nasopharynx in the form of biofilms. The biofilms act as bacterial reservoirs and planktonic bacteria from these biofilms can migrate to other sterile anatomical sites to cause pneumonia, otitis media (OM), bacteremia and meningitis. Human amniotic membrane contains numerous growth factors and antimicrobial activity; however, these have not been studied in detail. In this study, we prepared amniotic membrane extract and chorionic membrane extract (AME/CME) and evaluated their antibacterial and antibiofilm activities against *S. pneumoniae* using an *in vitro* biofilm model and *in vivo* OM rat model.

**Materials and Methods:** The AME/CME were prepared and protein was quantified using DC^TM^ (detergent compatible) method. The minimum inhibitory concentrations were determined using broth dilution method, and the synergistic effect of AME/CME with Penicillin-streptomycin was detected checkerboard. The *in vitro* biofilm and *in vivo* colonization of *S. pneumoniae* were studied using microtiter plate assay and OM rat model, respectively. The AME/CME-treated biofilms were examined using scanning electron microscope and confocal microscopy. To examine the constituents of AME/CME, we determined the proteins and peptides of AME/CME using tandem mass tag-based quantitative mass spectrometry.

**Results:** AME/CME treatment significantly (*p* < 0.05) inhibited *S. pneumoniae* growth in planktonic form and in biofilms. Combined application of AME/CME and Penicillin-streptomycin solution had a synergistic effect against *S. pneumoniae.* Biofilms grown with AME/CME were thin, scattered, and unorganized. AME/CME effectively eradicated pre-established pneumococci biofilms and has a bactericidal effect. AME treatment significantly (*p* < 0.05) reduced bacterial colonization in the rat middle ear. The proteomics analysis revealed that the AME/CME contains hydrolase, ribonuclease, protease, and other antimicrobial proteins and peptides.

**Conclusion:** AME/CME inhibits *S. pneumoniae* growth in the planktonic and biofilm states via its antimicrobial proteins and peptides. AME/CME are non-cytotoxic, natural human product; therefore, they may be used alone or with antibiotics to treat *S. pneumoniae* infections.

## Introduction

*Streptococcus pneumoniae*, is a Gram-positive bacterium that causes various diseases in humans and is responsible for 1 million deaths annually (O’Brien et al., 2009). *S. pneumoniae* colonizes the human nasopharynx asymptomatically, typically by 2 years of age (Gray et al., 1982), and the transfer of these pneumococci to sterile sites in the body can cause disease. *S. pneumoniae* causes various diseases such as bacterial meningitis, pneumonia, otitis media (OM), sinusitis, and conjunctivitis in children, the elderly, and the immune-compressed (Musher, 1992; Lieberthal et al., 2013). Pneumococci initially colonize the human nasopharynx in the form of specialized structures called biofilms (Bogaert et al., 2004; Simell et al., 2012). Biofilms are defined as organized communities of aggregated cells embedded in a hydrated matrix of extracellular polymeric substances (EPS) (Donlan and Costerton, 2002). Biofilms act as bacterial reservoirs, and planktonic bacteria from these biofilms can migrate to other sterile anatomical sites to cause pneumonia and OM (Hall-Stoodley et al., 2006; Sanchez et al., 2010; Weimer et al., 2010). They can also migrate to the blood stream, causing bacteremia, or to the brain, causing meningitis (Ash and Sheffield, 2013; Pichichero, 2013; Shak et al., 2013). Indeed, pneumococcal biofilms have been detected in human sinus mucosa biopsies and resected adenoids from individuals with tonsillitis, and have been observed in tympanostomy tubes collected from children with chronic OM (Hall-Stoodley et al., 2006; Hoa et al., 2009). Moreover, chronic wound infections, skin infections, burn injuries, and ophthalmic infections are also sites of bacterial biofilm formation (Attinger and Wolcott, 2012; Bispo et al., 2015; Percival et al., 2015; Ramos-Gallardo, 2016; Fleming et al., 2017). In addition, pneumococcal biofilms have been observed in the middle ears of experimentally infected chinchillas with *S. pneumoniae*, as well as bronchial and nasal lavage fluids obtained from the nasopharynxes of infected mice (Reid et al., 2009; Sanchez et al., 2010). It has been suggested that the bacteria within biofilms are embedded in a self-produced matrix of EPS and are thus resistant to both antibiotics and host immune defenses (Costerton et al., 1995; Donlan and Costerton, 2002). The increased resistance of biofilms to antimicrobial agents could be caused by, for example, mutations in bacteria affecting the drug target, the presence of efflux pumps or the production of modifying enzymes. In addition, the biofilm structure may obstruct the penetration of the antimicrobial agents because; (a) the charged polymers of the biofilm matrix might hamper drug diffusion, or (b) the biofilm matrix could restrict the antimicrobial agents to the surface of biofilms, leaving more deeply embedded cells relatively unaffected (Marsh, 2004). Bacteria growing under biofilms display a novel phenotype and could adapt to an alternative metabolic pathway compared with their planktonic counterparts; therefore, biofilm bacteria could become less sensitive to many antimicrobials such as beta-lactams and quinolones, which target bacterial macromolecule synthesis or metabolic pathways (Xie et al., 2005; Mascio et al., 2007). Bacteria in mature biofilms grow slowly under nutrient depleted conditions that make them markedly less susceptible to antimicrobial agents compared with fast growing cells (Davies, 2003). The emergence of antibiotic-resistant strains further hampers treatment options. Consequently, there is urgent need to discover new classes of antimicrobials or anti-biofilm agents that are safe and that limit the emergence of antibiotic-resistant bacteria (Hurdle et al., 2011). One strategy is to identify innate antimicrobial agents of animals and plants with multiple targets that are unlikely to evoke bacterial resistance (Zasloff, 2002; Costa et al., 2006; Hancock and Sahl, 2006). Various naturally occurring antimicrobial agents derived from plants have been reported as effective in controlling planktonic, as well as biofilm, bacteria (Kwieciński et al., 2009; Ma et al., 2012; Quave et al., 2012; Burt et al., 2014; Yadav et al., 2015). In addition, many antimicrobials have been identified in humans and other animals (Hancock and Sahl, 2006; Noore et al., 2013; Zhang et al., 2015; Coorens et al., 2017).

The human amniotic sac comprises a pair of avascular membranes that holds the developing embryo. The inner fetal membrane that encloses the amniotic cavity, containing the amniotic fluid and the fetus, is called the amnion, and the outer membrane is called the chorion, which contains the amnion and is part of the placenta. The human amniotic membrane exhibits anti-scarring, anti-inflammatory, immune-regulatory anti-fibrotic activities (Faulk et al., 1980; Hao et al., 2000; Bailo et al., 2004; Ricci et al., 2013; Silini et al., 2015). And the amniotic and chorionic membrane antimicrobial activity has been previously reported (Inge et al., 1991; Kjaergaard et al., 2001; Sangwan and Basu, 2010; Parthasarathy et al., 2014). These properties make the amniotic membrane an excellent biomaterial for the application of therapeutics for skin, including burn, injuries, ophthalmic surgery, and wound healing (Mamede et al., 2012; Gheorghe et al., 2016; Mohan et al., 2017). However, the application of amniotic membrane in a clinical setting has been hampered because of the need to preserve and store the whole membrane. More-over, the antibiofilm activity of amniotic and chorionic membrane extracts is not known. Therefore, in this study, we prepared amniotic membrane extracts and chorionic membrane extracts (AME/CME) and evaluated their antibacterial and antibiofilm activities against *S. pneumoniae* using an *in vitro* biofilm model and *in vivo* OM rat model.

## Materials and Methods

### Ethics Statement

Human amniotic membranes were collected from the Gynecology Department, Korea University Guro Hospital, Seoul, South Korea. Prior written consent was obtained from each volunteer donor, and the samples were collected after caesarian sections. The animal experimental protocols were performed according to the guidelines of the ethics committee of Korea University, Guro Hospital, and all the protocols followed in this study were approved by the Institute Review Board (IRB) of Korea University, Guro Hospital, Seoul, South Korea (approval number UGH16046002).

### Bacteria Strains and Growth Conditions

*Streptococcus pneumoniae* D-39 (NCTC 7466) is Avery’s Virulent, Serotype 2 encapsulated Strain, was purchased from the Health Protection Agency Culture Collections (HPA, Salisbury, United Kingdom). It is fully sequenced (Lanie et al., 2007), forms robust *in vitro* biofilms (Vidal et al., 2011) and it remained extremely virulent in animal models of infections after many year of its isolation form patient (Benton et al., 1995; Blue et al., 2003; LeMessurier et al., 2006). *S. pneumoniae* serotype 3 (ATCC strain 6303), 19A, and 19F (ATCC strain 49619) were purchased from the American Type Culture Collection (Manassas, VA, United States); serotype 11 (strain 7101975) was obtained from the Infectious Disease Department of Korea University Medical Center, Guro Hospital, Seoul. *S. pneumoniae* strains were grown on brain heart infusion (BHI) broth medium or on blood agar plates (BAPs) supplemented with 5% v/v sheep blood. The BAPs were pre-made and purchased from Shin Yang Chemicals Co., Ltd. (Seoul, South Korea). For minimum inhibitory concentrations (MICs) detection bacteria were grown in Muller-Hinton broth and agar (Sigma, St. Louis, MO, United States).

### AME/CME Preparation

The human AM matrices were collected from the Gynecology Department, Korea University Guro Hospital, Seoul, South Korea. Before the membrane samples were collected, the prospective donors were examined for gestational diabetes, pre-eclampsia, or infectious disease. Those testing positive for any of the above were excluded from the study. AME/CME were prepared using methods described previously by He et al. (2008) with some modifications reported in our previous studies (Go et al., 2016, 2017). Briefly, the tissues were washed with phosphate-buffered saline (PBS) three times to remove blood clots, and then sliced into small pieces, frozen using liquid nitrogen and ground into fine particles using a mortar and pestle. The membrane particles were then mixed with PBS at ratio of 1:1 (wt/vol) and homogenized on ice for 1 h. The membrane lysates were centrifuged twice at 12000 rpm at 4°C for 10 min to obtain the supernatants. The supernatants were filter (0.22-μm pore size) sterilized. The protein concentrations in the extracts were determined using a Detergent Compatible (DC) Protein Assay (Bio-Rad, Hercules, CA, United States) according to the manufacturer’s instructions. Briefly, 5 μL each of AME/CME extract (unknown) and standard solutions (from 2 to 0.125 mg/mL) were inoculated in triplicate in a 96-well plate. Solution A (25 μL; supplied with kit) was added to each well followed by 200 μL of solution B. The plates were incubated at room temperature for 15 min and absorbance was measured at 750 nm using microplate-reader. The standard curve was constructed and concentration of AME/CME extracts was calculated. The extracts were stored at -80°C until use.

### MIC Detection

Minimum inhibitory concentrations were determined using the broth micro-dilution method as recommended by the Clinical and Laboratory Standards Institute (CLSI, 2005). Overnight cultures of bacteria in MH broth (MHB) were diluted (1:10) in fresh medium and grown to the exponential phase. From the cells growing at the exponential phase, cell suspensions of 1–5 × 10^5^ colony-forming units (cfu)/mL were prepared. Serial twofold dilutions of AME/CME, from 2 to 512 μg, were added to the MHB cell suspensions. Thereafter 200 μL of each suspension were inoculated into each well of a 96-well plate. Negative control wells were inoculated with cell suspension only. The plates were incubated for 24 h at 37°C, and bacterial growth was determined by measuring the optical density at 600 nm (OD_600_) using a microplate reader. To further confirm the bacterial growth in each well after incubation, 10-μL aliquots of the cell suspensions from the 96-well plate were spread on MH agar, and bacterial colonies were counted after 24 h of incubation at 37°C. The MIC was defined as the lowest AME/CME concentration in which no visible growth was observed. The experiment was performed in quadruplicates and was repeated two times.

### Effect of AME/CME on *in Vitro* Biofilm Growth of *S. pneumoniae*

In this study, biofilm experiments were performed using static biofilm model, with 18 h biofilm growth as per pervious reports (Moscoso et al., 2006; Domenech et al., 2013). A microtiter plate assay was used to grow and quantify *in vitro* biofilms of the bacteria tested with or without AME/CME extracts (Christensen et al., 1985; Yadav et al., 2017). Briefly, the biofilms were grown to stationary phase in 24- or 96-well polystyrene flat-bottom microtiter plates (BD Falcon, Sparks, MD, United States) for 18 h at 37°C. Fresh bacterial cell suspensions were prepared in BHI medium, diluted 1:200, and inoculated into plates at 200 μL/well in 96-well plates or 1 mL/well in 24-well plates. Twofold diluted AME/CME from 16 to 256 μg, was added to the wells and incubated at 37°C for 18 h. PBS alone was introduced into the vehicle control wells. After inoculation, the media and planktonic bacteria cultures were decanted, washed twice with sterile water, and stained with 50 μl of 0.1% crystal violet (CV) for 15 min. After staining, the plates were washed and dried, and CV was dissolved in 200 μL (96-well plates) or 1 mL (24-well plates) of ethanol for each well. Absorbance at 570 nm was then measured using a microplate reader.

The metabolically active cells in the biofilms were detected using resazurin staining. Resazurin is a blue, non-fluorescent dye that is reduced to the pink and highly fluorescent compound resorufin by metabolically active cells, thus allowing the quantitative measurement of cell viability. Resazurin staining was performed using a previously reported procedure with minor changes (Paytubi et al., 2017). Briefly, 0.02% (w/v) resazurin sodium salt (Sigma) solution was prepared in water and sterilized by filtration. Biofilm were growth in 96-well microtiter plates with different concentration of AME/CME (16–256 μg) for 18 h. The biofilms were washed twice with sterile water and incubated with 25 μL (0.02%) resazurin dye and 100 μL BHI-broth. The plate was incubated at 37°C for 2 h and fluorescence was measured (excitation 570 nm, emission 615 nm) using a Varioskan LUX multimode microplate reader (Thermo Scientific, Waltham, MA, United States).

### Effect of AME/CME on Established *S. pneumoniae* Biofilms

Eighteen-hour pre-established biofilms of *S. pneumoniae* strain D-39 were treated with two and fourfold MICs of AME/CME and incubated for an addition 6 h. The biofilm biomass was assessed by using the CV microplate assay, and viable bacteria were enumerated and presented as cfu counts, as described above.

### Determination of the Bacterial Killing Rate

*Streptococcus pneumoniae* strain D-39 was grown to the log phase, and aliquots were removed, treated with 512 μg (2 × MIC) AME or CME, and further incubated. The bacteria were counted at various time intervals (0, 1, 3, and 6 h). The controls were treated with PBS.

### Synergistic Effects of AME/CME and Penicillin-Streptomycin (P-S) Antibiotic Solution on *S. pneumoniae*

Penicillin-Streptomycin antibiotic solution is commonly used for washing AMs before preservation to eliminate possible microbial contamination. In this study, we evaluated the synergistic effects of AME/CME and P-S antibiotic solution on *S. pneumoniae* by using the checkerboard test, as previously described (Petersen et al., 2006). *S. pneumoniae* D-39 was grown with twofold dilutions from 32 μg (0.125 × MIC) to 256 μg (MIC) of AME/CME, as well as 0.025 μg (0.125 × MIC) to 0.2 μg (MIC) of P-S antibiotic solution alone and in combination. In each well of a 96-well plate, the AME/CME or antibiotic solution concentrations were adjusted such that the first antibiotic of the combination was serially diluted along the ordinate, while the second drug was diluted along the abscissa. Growth was monitored by determining the optical density at 600 nm. The fractional inhibitory concentration index (FICI) was calculated as the sum of the fractional inhibitory concentrations (FICs) of the AME/CME and P-S antibiotic solution. FICs are defined as the MIC of each drug when used in combination, divided by the MIC of the drug when used alone. If the FICI is less than or equal to 0.5, the interaction of the two drugs is synergistic. The interaction is additive if the FICI > 0.5 and ≤1. The interaction is indifferent if the FICI is >1.0 and ≤2.0, and antagonistic if the FICI is >2.0.

To evaluate the effect of AME or CME+ P-S antibiotic solution (AME/CME solution) on *S. pneumoniae* biofilm, *in vitro* biofilms were grown with different concentration of AME/CME solution (4.003–64.05 μg) and biofilm biomass was quantified using the CV-microtiter plate assay as described above. The metabolically active cells within biofilms were detected using resazurin staining as described above. To evaluate the biofilm eradication potential of AME or CME + P-S antibiotic solution, 18 h pre-established biofilms of *S. pneumoniae* D-39 were treated with AME or CME + P-S antibiotic solution (2 × MIC and 4 × MIC) and incubated for an additional 6 h. The biofilm biomass was assessed by CV microplate assay, and viable bacteria were detected by resazurin staining as described above.

### Scanning Electron Microscope Analysis of Biofilm Eradication

It is reported that *S. pneumoniae in vitro* biofilms formed on abiotic surfaces for 10–12 h consist of a three-dimensional structures with significant thickness (25–30 μm) (Moscoso et al., 2006). In this study, we evaluated the effect of AME/CME on 18-h pre-established *in vitro* biofilm using scanning electron microscope (SEM). *In vitro* biofilms of *S. pneumoniae* D-39 were grown as described above for 18 h and treated with indicated concentration of AME/CME solution for 6 h; the control biofilms were treated with PBS. After incubation, the biofilms were washed, pre-fixed by immersion in 2% glutaraldehyde in 0.1 M phosphate buffer, and post-fixed for 2 h in 1% osmic acid dissolved in PBS. The biofilm samples were then treated with increasing concentrations of ethanol (60–95%), followed by t-butyl alcohol. The samples were dried in a freeze dryer (ES-2030, Hitachi, Tokyo, Japan) and coated with platinum using an ion coater (IB-5, Eiko, Kanagawa, Japan). The images were captured under field emission-SEM (FE-SEM; Hitachi, S-4700, Tokyo, Japan).

### Confocal Microscopy of Biofilms Treated with AME/CME

The biofilm eradication activity of AME/CME was evaluated by confocal microscopy. The biofilms were grown on micro-discs for 18 h and then treated with AME/CME solution (2 × MIC) for 6 h. The control biofilms were treated with PBS only. The biofilms were stained using a LIVE/DEAD Biofilm Viability Kit (Invitrogen) using procedures described by the manufacturer. Control cells were grown without AME/CME solution treatment. After washing, the stained biofilms were viewed under a Nikon A1 confocal microscope (Nikon Instruments Inc., Melville, NY, United States) using fluorescein (green) and Texas red (red) bandpass filter sets.

### Effect of AME on *in Vivo* Colonization of *S. pneumoniae* in the Middle Ears of Rats

The inhibitory effect of AME on the *in vivo* colonization of *S. pneumoniae* was evaluated using a rat OM model (Yadav et al., 2012, 2017). Twenty-six pathogen-free Sprague-Dawley rats weighing 150–200 g were obtained from Koatech (Pyeongtaek, South Korea). The rats were isolated in a sterile environment for 2 weeks, and middle ear abnormalities were examined before the experiment. The rats were divided into four groups: group 1 rats (*n* = 9) were inoculated with bacteria only; group 2 rats (*n* = 9) were inoculated with bacteria and AME solution (0.5 × MIC); group 3 rats (*n* = 6) received medium only (vehicle control); and group 4 rats (*n* = 3) had no procedures performed. Animals were euthanized using Zoletil H (tiletamine-zolazepam, Virbac, Carros, France) and Rompun H (xylazine-hydrochloride, Bayer, Leverkusen, Germany) combined at a ratio of 1:1. The *S. pneumoniae* cell suspension was prepared in BHI medium, and 50 μL (approximately 10^7^ cfu) was injected into the middle ear cavity of each rat in groups 1 and 2 through the tympanic membrane of the right ear using a tuberculin syringe and 27-gauge needle (Yadav et al., 2012). The animals were held for 1 week before they were sacrificed and bullae were acquired aseptically. Tympanic membranes were removed, and the middle ears were dissected and photographed. The representative bullae from rats in each group were preserved in SEM solution (glutaraldehyde and paraformaldehyde solution), aseptically homogenized, serially diluted, and plated on BAP for cfu counts. Colonies were counted after 24 h of incubation at 37°C.

### AME/CME Proteomics Analysis

A proteomic analysis of AME/CME was performed using tandem mass tag (TMT)-based quantitative mass spectrometry (MS) using previously described procedures (Park et al., 2016). Briefly, the extracts were denatured with 8 M urea at room temperature for 10 min. The e protein solution was then reduced using dithiothreitol (DTT) at room temperature and alkylated using indole acetic acid (IAA). Proteins were quantified using the Bradford protein assay, using 100-μg aliquots of protein diluted in 50 mM ABC buffer. The diluted protein samples were digested with trypsin at 37°C for 16 h and quenched in 3% FA The digested samples were dissolved in mobile phase A and analyzed using a liquid chromatography (LC)-MS/MS system consisting of a nano liquid chromatograph and a mass spectrometer equipped with a nano electrospray source. The samples were loaded into a C_18_ trap column with an autosampler. Samples were concentrated and desalted on the trap column for 5–10 min at a flow rate of several μL/min and then separated on a C_18_ analytical column. For the LC–MS/MS analysis, the mobile phases comprised 99.9% water (A) and 99.9% acetonitrile (ACN) (B), with each containing 0.1% FA. The LC gradient started with 5% of B for 15 min and was ramped to 15% of B for 5 min, 50% of B for 75 min, and 95% of B for 1 min; it was held at 95% of B for 13 min and then at 5% of B for additional 1 min. The column was re-equilibrated with 5% of B for 10 min before the next run. For the serum/plasma sample analysis, the LC gradient time was extended to 180 min. For peptide identification, MASCOT (version 2.4.0, Matrix Science, London, United Kingdom) software was used. Peptides with a *p*-value < 0.05 (automatically estimated by MASCOT) were considered authentic.

### Statistical Analysis

Experiments were performed in quadruplicates and repeated 2–3 times. The initial comparisons were performed with non-parametric Wilcoxon method. The difference in value between control and treated samples were assessed with the Mann–Whitney *U* tests and represented as median values and quartiles (25/75%). Comparisons with a *p*-value ≤ 0.05 were considered statistically significant. Statistical analyses were performed using SPSS Statistics package (SPSS Inc., Chicago, IL, United States).

## Results

### MICs for *S. pneumoniae*

For MIC detection *S. pneumoniae* D-39, serotype 3, 19A, 19F, and clinical strain 11 were grown with different concentrations of AME/CME (2–512 μg). Bacterial growth was detected by measuring absorbance at 600 nm. The bacteria growth were further confirmed by spreading 10 μL samples from each well on MH agar plate. The results showed no growth of *S. pneumoniae* D-39 and serotype 3 at AME/CME concentrations of 256 μg and above. Similarly, the growth of *S. pneumoniae* serotypes 19A and 19F were inhibited at 128 μg of AME/CME. The clinical strain 11 showed no growth at 32 μg of AME/CME. Therefore, the MICs of AME and CME for *S. pneumoniae* D-39, *S. pneumoniae* serotype 3, 19A, 19F and clinical strain 11 were 256, 256, 128, 128, and 32 μg, respectively.

### AME/CME Inhibited *in Vitro* Biofilm Growth of *S. pneumoniae*

**FIGURE 1:**
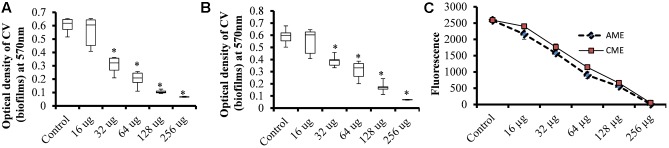
*In vitro* biofilm growth of *Streptococcus pneumoniae* D-39 with various concentrations of amniotic membrane extract and chorionic membrane extract (AME/CME). Biomasses of biofilms grown with different concentration of **(A)** AME and **(B)** CME were assessed using a crystal-violet microtiter plate assay. **(C)** Metabolically active cells within biofilms grown with AME/CME were detected using resazurin staining. Statistical analyses were performed using the Mann–Whitney *U* tests and represented as median values and quartiles (25/75%). Comparisons with a *p*-value (^∗^*p* < 0.05) were considered statistically significant.

To assess the effect of AME/CME on *S. pneumonia* biofilm growth *in vitro*, biofilms were grown on microtiter plates with different concentration of AME/CME. The CV microtiter plate assay results revealed a significant (*p* < 0.05) decrease in biofilm biomass in AME/CME-supplemented samples (**Figures 1A,B**). The inhibitory effect of AME/CME on pneumococcal biofilms was concentration dependent. At sub-MIC (128 μg) concentrations of the AME/CME supplement, the pneumococci were unable to form robust biofilms, and the biomass decreased by >50%. Similarly, the viable bacteria detected by resazurin staining also decreased with increasing concentrations of AME/CME (**Figure 1C**). The bacteria appeared unable to grow in the presence of AME/CME, and were not able to establish organized biofilms. These results indicate that AME/CME inhibits *in vitro* biofilm formation.

### AME/CME Effectively Eradicated Pre-established *S. pneumoniae* Biofilms *in Vitro*

**FIGURE 2:**
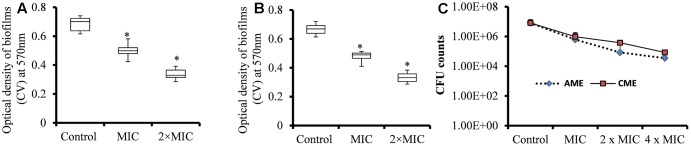
Biomass quantification and cfu counts of *S. pneumoniae* D-39 pre-established biofilms treated with different concentrations of AME/CME. **(A)** Quantification of the biomass of *S. pneumoniae* biofilms treated with 256 μg (MIC) and 512 μg (2 × MIC) of AME and **(B)** CME. **(C)** Count of viable bacteria in *S. pneumoniae* biofilms treated with 256 μg (MIC), 512 μg (2 × MIC) and 1024 μg (4 × MIC) of AME/CME. Statistical analyses of biofilm biomasses were performed using the Mann–Whitney *U* tests and represented as median values and quartiles (25/75%). Comparisons with a *p*-value (^∗^*p* < 0.05) were considered statistically significant.

The EPS matrices of pneumococcal biofilms contain proteins, e-extracellular DNA, and polysaccharides, and are difficult to eradicate using conventional antibiotics (Moscoso et al., 2006). We evaluated the ability of AME/CME to eradicate pre-established *S. pneumoniae* biofilms. The results showed strong biofilm eradication activity of AME/CME against pneumococcal biofilms. The biomasses of pre-established pneumococcal biofilms was significantly (*p* < 0.05) reduced by AME/CME treatment at the MIC (256 μg) and 2 × MIC (512 μg) (**Figures 2A,B**). Treatment with 2 × MIC (512 μg) AME/CME significantly (*p* < 0.05) reduced *in vitro* biofilms > 50%. Similarly, the viable bacteria counts within biofilms also decreased significantly after 6 h of incubation with 256 μg (MIC), 512 μg (2 × MIC) and 1024 μg (4 × MIC) of AME/CME (**Figure 2C**). AME/CME treatment at 4 × MIC significantly (*p* < 0.05) decreased viable bacteria by more than 2 log 10 steps.

**FIGURE 3:**
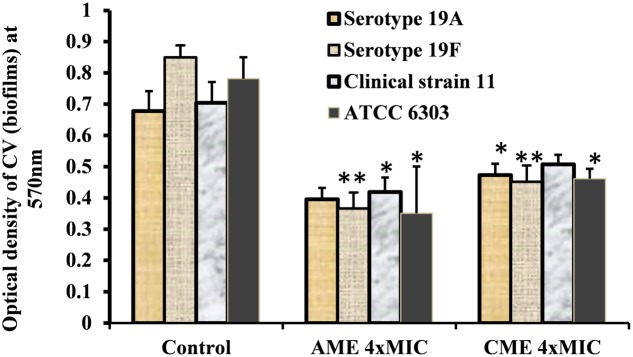
*In vitro* pre-established biofilms of *S. pneumonia* clinical strains eradicated by 1024 μg (4 × MIC) of AME/CME. Error bars represent standard deviations from the mean. Comparisons with a *p*-value (^∗^*p* < 0.05) were considered statistically significant.

Strong biofilm eradication activity of AME/CME at concentration of 4 × MIC (1024 μg) against clinical strains of *S. pneumoniae* was also detected (**Figure 3**). These results indicated that AME and CME possess strong pneumococci biofilm eradication activity.

### AME/CME Showed Strong Bactericidal Activity

**FIGURE 4:**
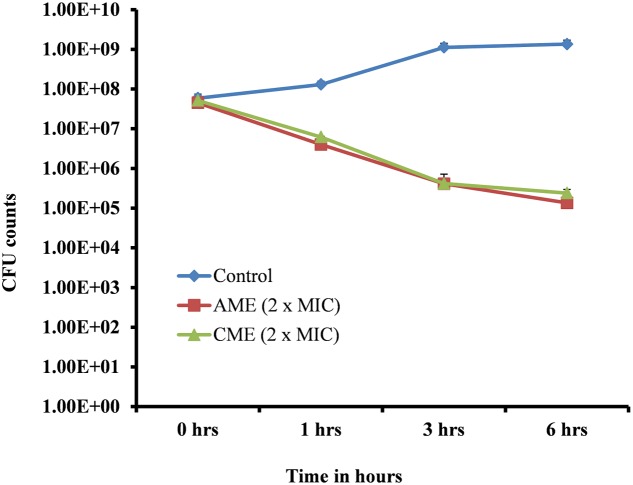
Time-killing analysis of *S. pneumoniae* D-39. Cells in the exponential growth phase were treated with 512 μg (2 × MIC) AME/CME and viable bacteria were assessed by cfu counts at various time points (0, 1, 3, and 6 h).

Amniotic membrane extract and chorionic membrane extract is a natural cell membrane extract from the human amnion; therefore, these extracts were expected to show significant bacteriostatic effects. We sought to test the bactericidal effects of AME/CME on pneumococci. A time-kill analysis was performed on bacteria in the exponential growth phase. Treatment of log phase bacteria with 512 μg (2 × MIC) of the extracts exhibited a strong bactericidal effect on *S. pneumoniae*. At 0 h, viable bacteria in the control and treated samples were similar (5.15 × 10^7^); however, 1 h after AME/CME treatment, the viable bacteria decreased significantly (to 6.20 × 10^6^). The viability of bacteria continued to decrease with incubation time, and at 3 and 6 h of AME/CME treatment, viable bacteria were reduced by more than two and three logs, respectively (**Figure 4**). These results demonstrated the bactericidal activity of AME/CME against pneumococci.

### Synergistic Effects of AME/CME and Penicillin-Streptomycin (P-S) Antibiotic Solution on *S. pneumoniae*

**FIGURE 5:**
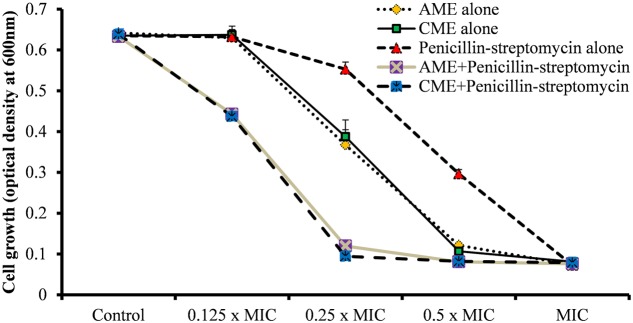
Synergistic effects of AME/CME and Penicillin-streptomycin solution on *S. pneumoniae* D-39. Bacteria were grown in presence of two-fold dilutions of AME/CME from 32 μg (0.125 × MIC) to 256 μg (MIC) or the antibiotic solution from 0.025 μg (0.125 × MIC) to 0.2 μg (MIC) alone and in combination. The growth was determined by the measuring optical density at 600 nm.

*Streptococcus pneumoniae* growth was inhibited by 256 μg AME or CME. Similarly, the P-S antibiotic solution at a concentration of 0.2 μg completely inhibited bacterial growth. Therefore, the individual MICs of AME or CME and the P-S antibiotic solution on *S. pneumoniae* were 256 and 0.2 μg, respectively. In combination (AME+ P-S antibiotic or CME+ P-S antibiotic), the MICs of AME or CME and P-S antibiotic solution decreased to 64 and 0.05 μg, respectively. No bacterial growth inhibition was detected with AME or CME alone at a concentration of 64 μg (0.25 × MIC). Similarly, no growth inhibition was detected with 0.05 μg (0.25 × MIC) of P-S antibiotic solution when applied alone. However, the bacterial growth was inhibited by combining AME or CME at concentration of 64 μg (0.25 × MIC) and P-S antibiotic solution at concentration of 0.05 μg (0.25 × MIC) (**Figure 5**). According to the definition of FICs, the FIC value of AME or CME was 0.25 [MIC in combination (64 μg)/individual MIC (256 μg). Similarly, the FIC value of P-S antibiotic solution was 0.25 (0.05 μg/0.2 μg). Therefore, the FICI of AME or CME and P-S antibiotic solution in combination was 0.5, indicating a synergistic effect (as defined by an FICI ≤ 0.5). This result implied that in combination AME or CME and P-S antibiotic solution (AME/CME solution) exhibited a synergistic effect against *S. pneumoniae*.

**FIGURE 6:**
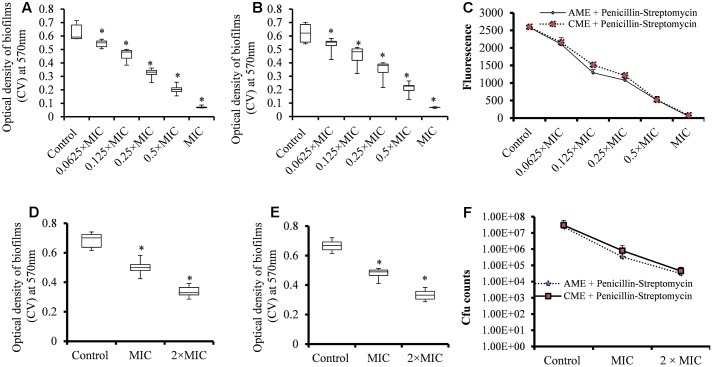
Effect of AME/CME + Penicillin-streptomycin solution on *S. pneumoniae* biofilm growth and eradication of pre-established biofilms. **(A)** Quantification of biofilms biomass grown with different concentrations of AME+Penicillin-streptomycin solution. **(B)** Quantification of biofilms biomass grown with different concentrations of CME+Penicillin-streptomycin solution. **(C)** Metabolically active cells within biofilms grown with different concentrations of AME/CME were detected using resazurin staining. **(D)** Quantification of the biomass of pre-established biofilms treated with (MIC and 2 × MIC) of AME+Penicillin-streptomycin solution, or **(E)** CME+Penicillin-streptomycin solution. **(F)** Cfu counts of pre-established biofilms treated with AME/CME+Penicillin-streptomycin solution (MIC and 2 × MIC). Statistical analyses of biofilm biomasses were performed using the Mann–Whitney *U* tests and represented as median values and quartiles (25/75%). Comparisons with a *p*-value (^∗^*p* < 0.05) were considered statistically significant.

The synergistic experiment results showed that the AME or CME in combination with P-S antibiotic solutions inhibited bacterial growth at low concentration. Therefore, we prepared AME or CME solution by combining AME or CME and Penicillin-streptomycin solution and evaluated biofilm inhibition and biofilm eradication activity. The results showed that increasing concentrations of the AME+P-S or CME+P-S solution significantly (*p* < 0.05) decreased biofilm growth *in vitro*. A significant (*p* < 0.05) reduced biofilm biomass and viable bacteria were detected at 4.003 μg (0.0625 × MIC) of AME/CME+P-S solutions and the inhibitory effect of AME/CME+P-S solutions was concentration dependent (**Figures 6A–C**). The AME/CME+P-S solution was also effective to eradicate pre-established biofilms of *S. pneumoniae*. Treatment of pre-established biofilm with 64.05 μg (MIC) and 128.1 μg (2 × MIC) of AME/CME+P-S solutions significantly (*p* < 0.05) reduced biofilm biomass (**Figures 6D,E**). Similarly, viable bacteria were also decreased in AME or CME+P-S solution treated biofilms (**Figure 6F**). These results indicate that the AME/CME+P-S solution is an effective antimicrobial solution for *S. pneumoniae* biofilms.

### Confocal Microscopy Analysis of AME/CME Solution Treated Biofilms

**FIGURE 7:**
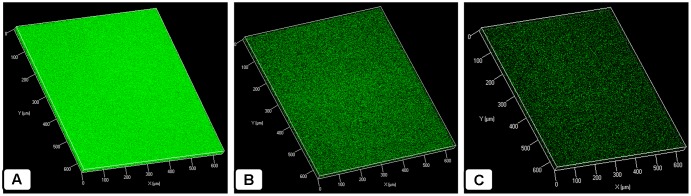
Confocal microscopic analysis of *in vitro S. pneumoniae* D-39 biofilms treated with AME/CME + Penicillin-streptomycin solution for 6 h. **(A)** Control biofilm treated with PBS. **(B)** Biofilms treated with 2 × MIC AME. **(C)** Biofilms treated with 2 × MIC CME.

The confocal microscopy results showed that the untreated biofilms were compact and multilayered and the cells were connected to each other to form an organized three-dimensional structure (**Figure 7A**). However, the biofilms treated with AME/CME solution were scattered and attached to the bottom of the plate (**Figures 7B,C**). The cell-to-cell connections were not well established, and the biofilms were dispersed.

### SEM Analysis of AME/CME Solution-Treated Biofilms

**FIGURE 8:**
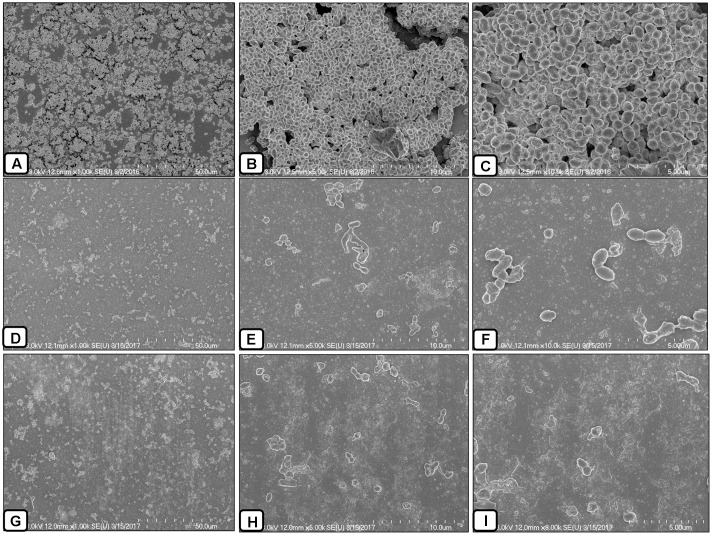
Scanning electron microscope (SEM) analysis of pre-established biofilms of *S. pneumoniae* D-39 treated with 2 × MIC of AME/CME solution. **(A–C)** Are SEM images of PBS-treated biofilms (control). **(D–F)** Are SEM images of AME solution treated biofilms. **(G–I)** Are SEM images of CME solution treated biofilms. Images are 5 and 10 μm.

In this study, the SEM analysis showed compact biofilms in the control samples. The cells were well connected to each other and the bottom of the plate, forming three-dimensional biofilms (**Figures 8A–C**). The cells in the biofilms produced extracellular polymeric substances that were visible on and between the cells. Conversely, the biofilms treated with AME/CME solution were scattered, irregular, and disrupted (**Figures 8D–I**). In these biofilms, only a few cells that were scattered on the bottom of the plate were visible. The cell-to-cell connections were not well established, and the biofilms were disorganized. AME/CME solution treatment appeared to disrupt cell-to-cell connections and dismantle biofilm matrices. These results provided further evidence that AME/CME demonstrates a potent biofilm eradication activity.

### AME Solution Reduced *in Vivo* Colonization by *S. pneumoniae*

**FIGURE 9:**
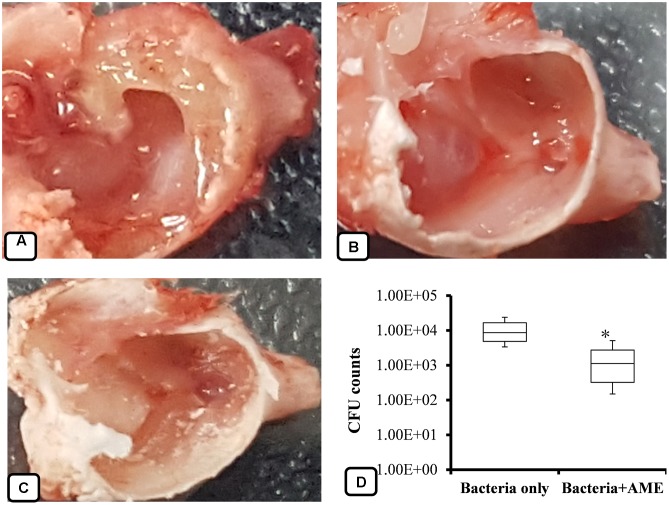
Effect of AME solution on *in vivo* colonization by *S. pneumoniae* in the middle ears of rats. **(A)** Rat bulla inoculated with bacteria only. **(B)** A rat bulla inoculated with bacteria and AME. **(C)** A rat bulla inoculated with medium only. **(D)** Counts of bacteria from the middle ears of rats 1 week after inoculation. Statistical analyses were performed using the Mann–Whitney *U* tests and represented as median values and quartiles (25/75%). Comparisons with a *p*-value (^∗^*p* < 0.05) were considered statistically significant.

The inhibitory effects of AME solution on *in vivo* colonization was studied using a rat OM model. *S. pneumoniae* D-39 was inoculated into the middle ears of rats, and then bacteria were enumerated after 1 week and the mucosa was visualized under SEM. The results showed that rat bullae inoculated with bacteria only swelled and thickened; the bullae were filled with dense cell debris and biofilm-like material (**Figure 9A**). However, no visible cell-debris or biofilm-like structures were detected in the rat bullae inoculated with bacteria + AME solution (**Figure 9B**). Similarly, in vehicle control bullae, no biofilms or cell debris were detected and there was no mucosal swelling (**Figure 9C**). The mean cfu counts of bacteria in the middle ears inoculated with bacteria only and bacteria with AME were 1.13 × 10^4^ and 1.79 × 10^3^, respectively. In rat bulla inoculated with AME, significantly (*p* < 0.05) 84% less bacteria were recovered (**Figure 9D**).

### SEM Analysis of *in Vivo* Colonization by *S. pneumoniae*

**FIGURE 10:**
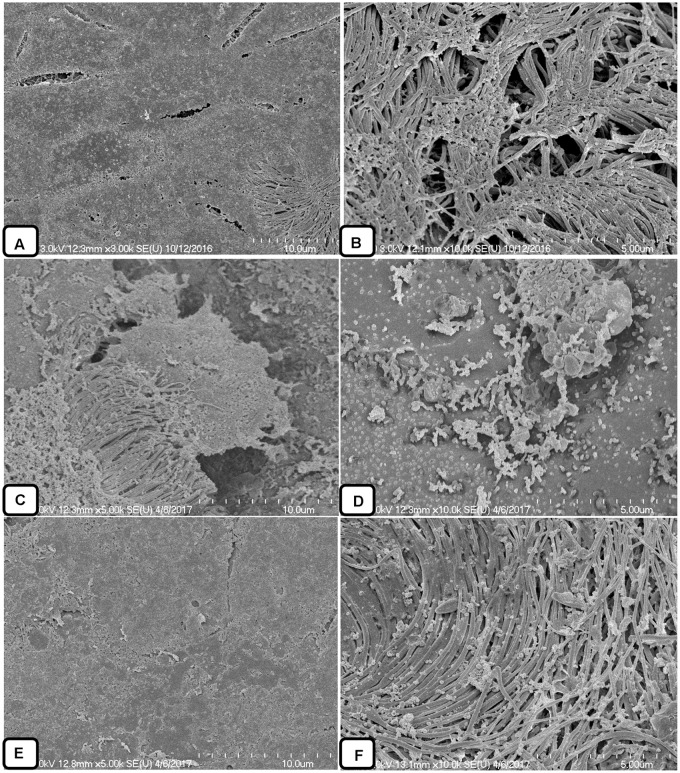
Scanning electron microscope analysis of the middle ears of rats inoculated with bacteria or bacteria with AME solution. **(A,B)** SEM images of middle ears inoculated with medium only. **(C,D)** SEM images of middle ears inoculated with bacteria only. **(E,F)** SEM images of middle ears inoculated with bacteria and AME.

The colonization of *S. pneumoniae* in the middle ear mucosa of rats was viewed using SEM (**Figure 10**). The middle ears treated with the vehicle control were clean, without any cell debris in the mucosa and with intact cilia (**Figures 10A,B**). The rat bullae inoculated with bacteria only were filled with biofilm-like structures, the mucosa was covered with biofilm debris, and cilia were not visible (**Figures 10C,D**). The biofilm EPS deposited on the tips of cilia concealed the cilia. In contrast, the rat bullae supplemented with AME solution were relatively clean, and there was no visible biofilm-like structure or cell debris on the middle ear mucosa. No visible cell debris was deposited on the cilia, although the cilia of the epithelium appeared conglomerated (**Figures 10E,F**).

### Proteomics Analysis

**FIGURE 11:**
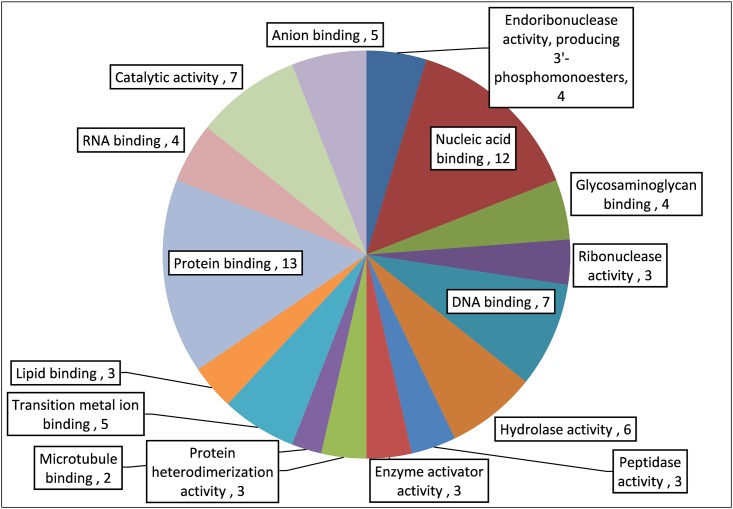
Gene ontology (GO) analysis of important antimicrobial proteins/peptides in AME/CME.

The proteomics analysis of AME/CME revealed several antimicrobial proteins/peptides (**Table 1**). The gene ontology (GO) analysis indicated that the identified proteins and peptides are involved in hydrolase, protease, and ribonuclease activities. The pathway analysis of these proteins revealed that, in humans, these proteins/peptides are also involved in chemotaxis and T cells and B cell activity in response to bacterial infection (**Figure 11**). The major protein/peptides included seven ribonucleases (ribonuclease T2, ribonuclease K6, ribonuclease 7, ribonuclease H2, ribonuclease pancreatic, and ribonuclease 5 [angiogenin]). The lactoferricins, which are antimicrobial peptides present in lactoferrin proteins, were also detected in AME/CME. A lysozyme with recognized muramidase or N-acetylmuramide glycanhydrolase activity was present in the AME/CME extracts. The other antimicrobial proteins and peptides present were dermcidin, a skin peptide, granulysin, the proteins S100-A9 and S100-A8, beta-2 microglobulin, and antileukoproteinase. The other proteins present in the extracts with antimicrobial activity were histone H2B type 1-D, type 1-O, HRA-V, and H1-4,and human beta defensin-3 protein.

**Table 1:** List of antimicrobial-active proteins/peptides present in amniotic membrane extract and chorionic membrane extract (AME/CME) extracts detected by tandem mass tag (TMT)-mass spectrometry (MS) spectroscopy.

Serial number	Accession number	Protein/peptide name	Peptide sequences
1	P61626	Lysozyme C	R.GISLANWMCLAK.W
2	O00584	Ribonuclease T2	K.LIMVQHWPETVCEK.I
3	Q93091	Ribonuclease K6	K.AHWFEIQHIQPSPLQCNR.A
4	Q9H1E1	Ribonuclease 7	K.GMTSSQWFK.I
5	Q8TDP1	Ribonuclease H2 subunit C	R.DSGTDDQEEEPLER.D
6	P07998	Ribonuclease pancreatic	R.QHMDSDSSPSSSSTYCNQMMR.R
7	P22749	Granulysin	R.TCLTIVQK.L
8	P81605	Dermcidin	K.ENAGEDPGLAR.Q
9	P03950	Angiogenin (RNase 5)	R.DDRYCESIMR.R
10	O00585	C-C motif chemokine 21	K.ELWVQQLMQHLDK.T
11	O95715	C-X-C motif chemokine 14	K.MVIITTK.S
12	P05067	Amyloid beta A4 protein	K.WDSDPSGTK.T
13	P02788	Lactoferricin	R.SVNGKEDAIWNLLR.Q
14	P05067	Amyloid beta A4 protein	K.WDSDPSGTK.T
15	P59666	Neutrophil defensin 3	R.IPACIAGER.R
16	P03973	Antileukoproteinase	K.AGVCPPK.K
17	P58876	Histone H2B type 1-D	R.KESYSVYVYK.V
18	P23527	Histone H2B type 1-O	R.KESYSIYVYK.V
19	Q71UI9	Histone H2A.V	R.AGLQFPVGR.I
20	P10412	Histone H1.4	R.KASGPPVSELITK.A
21	P06702	Protein S100-A9	R.NIETIINTFHQYSVK.L
22	P05109	Protein S100-A8	K.ALNSIIDVYHK.Y
23	P62158	Calmodulin	K.EAFSLFDKDGDGTITTK.E
24	P81605	Dermcidin	K.ENAGEDPGLAR.Q
25	P61769	Beta-2-microglobulin	K.IQVYSR.H


## Discussion

The human inner fetal membrane is the AM. It encloses the amniotic cavity, containing the amniotic fluid and the fetus. The outer membrane is called the chorion. It contains the amnion and is part of the placenta. The AM consist of a fetal component (the chorionic plate) and a maternal component (the decidua), and is composed of an epithelial monolayer, a thick basement membrane, and an avascular stroma. Blood vessels and nerves are absent, and nutrients are supplied directly. Many studies have demonstrated the antimicrobial activity of the AM (Inge et al., 1991; Kjaergaard et al., 2001); however, a detailed study of this activity has not been performed. The human AM is reported to possess antimicrobial activity (Kim et al., 2007; King et al., 2007), and the human amniotic/chorionic membrane has been used to dress incisions after surgery, for wound healing, for burn injuries, and in dentistry and ophthalmology (Mamede et al., 2012; Lafzi et al., 2016; Mohan et al., 2017). Wound infections, burn injuries, the oral cavity, and the eyes are common sites of bacterial infection and biofilm growth (Attinger and Wolcott, 2012; Akers et al., 2014; Bispo et al., 2015; Percival et al., 2015). Based on these previous studies, we hypothesized that AM/CM could be effective in controlling bacterial infection and biofilm growth. The AM/CM shows anti-angiogenic, pro-apoptotic, and immune-regulatory activity, reduces fibrosis, suppresses pro-inflammatory cytokines, stimulates epithelialization, and demonstrates antimicrobial activity. However, application of whole AM/CMs has been limited by the requirements to preserve these membranes, which are difficult to maintain. Therefore, in this study we prepared extracts of AM/CM and evaluated the antibacterial and anti-biofilm activity of AME/CME against *S. pneumoniae*.

The results of this study showed that AME or CME is effective in inhibiting pneumococcal biofilms *in vitro*. This effect on biofilms was concentration dependent. The primary reason for low aggregation of the bacteria in biofilms treated with AME/CME was inhibition of cell growth. However, the significant inhibition of biofilm biomass relative to that of planktonic cell growth indicated that the AME/CME intervened specifically in biofilm formation or that treated bacteria were loosely aggregated and unable to form organized biofilms. This anti-biofilm effect of AME/CME is important, especially with regards to the phenomenon of commonly used antibiotics causing bacteria to adapt to biofilm growth at sub-MIC concentrations (Blickwede et al., 2005; Hoffman et al., 2005; Li et al., 2005; Andersson and Hughes, 2014). Bacteria within biofilms are resistant to antibiotics, and high concentrations of antibiotics are required to eradicate established biofilms (Donlan and Costerton, 2002). In the present study, we demonstrated the significant biofilm eradication potential of AME/CME. It is likely that the AME/CME constituents dismantle the matrix and decrease the biofilm biomass. In addition, the low viability of treated biofilm could be caused by bactericidal constituents of AME/CME.

The P-S antibiotic solution is used to wash amniotic membranes during preservation to prevent microbial contamination (Laurent et al., 2014). Here we evaluated the synergist effect of AME/CME with P-S antibiotic solution. The results of this study showed that a combination of AME or CME with P-S antibiotic solution exhibited a synergistic effect against *S. pneumoniae*. AME or CME in combination with P-S antibiotic solution also significantly inhibited *in vitro* biofilm growth and eradicated pre-established biofilms. Moreover, the AME or CME+P-S antibiotics are effective on biofilms at low concentrations. The confocal microscopy analysis and SEM results further confirmed loose and scattered biofilms in AME/CME solution-treated biofilms. In addition, the low recovery of bacteria in rat middle ear inoculated with AME solution indicated that AME inhibited *in vivo* colonization by *S. pneumoniae*. The SEM analysis further confirmed the absence of biofilm-like structures in AME-treated rat middle ears. The decrease in biomass and low cfu counts indicated that AME/CME solution eradicated the biofilms by two mechanisms; first, these extracts disrupt cell–cell connections and dismantle the biofilms; second, they kill bacterial cells. AM/CM contains several antimicrobial peptides/proteins, including human neutrophil peptides 1, 2, and 3 (King et al., 2007), lysozyme (Yoshio et al., 2003), bactericidal/permeability-increasing protein (BPI) (Elsbach and Weiss, 1993), LL-37 (Yoshio et al., 2003), calprotectin (MRP8/14) (Lehrer, 1993), and ubiquitin (Kim et al., 2007). In addition, AM/CM also contain β3-defensin (Krisanaprakornkit et al., 1998; Buhimschi et al., 2004), secretory leukocyte proteinase inhibitor (SLPI), and elafin, which are expressed in the AM (Buhimschi et al., 2004; Kim et al., 2007). The anti-inflammatory elafin and SLPI both have antimicrobial properties and contribute to the innate immune response to protect against infection (King et al., 2003).

To examine the constituents of AME/CME, we determined the proteins and peptides of AME/CME using TMT-based quantitative MS. The proteomics analysis of AME/CME revealed hydrolase, ribonuclease, and other proteins and peptides with antimicrobial activities. The matrices of biofilms are most often composed of proteins, nucleic acids, exo-polysaccharides, and lipids (Domenech et al., 2013, 2016). It is likely that the AME/CME constituents such as lactoferricins, lysozyme, granulysin, defensin, dermcidin, ribonucleases and other proteins or peptides degraded biofilms components, leading to eradication of the biofilms. Previous studies have shown that extracellular DNA (eDNA) is an important component of the EPS in pneumococcal biofilms, and the exposure of the pneumococcal biofilms to nucleic acid degradative enzymes led to dispersion of the biofilms (Hall-Stoodley et al., 2006; Moscoso et al., 2006; Carrolo et al., 2010).

Lactoferrin is a mucosal glycoprotein present in milk, saliva, tears, semen, and neutrophil granules (Legrand et al., 2005). Lactoferrin is a multifunctional protein that exhibits bacteriostatic and antimicrobial activities against a wide variety of pathogens, including bacteria, virus, parasites, and fungi (Jenssen and Hancock, 2009). Lactoferricins are small 11-amino acid antimicrobial peptides derived from lactoferrin (Chapple et al., 1998). Lactoferricins contain one or more tryptophans and high concentrations of positively charged amino acid residues that attach to and destabilize negatively charged bacterial membranes (Wenzel et al., 2014). The bactericidal activity of lactoferricins against *S. pneumoniae* has been reported (Shaper et al., 2004). Furthermore, synergistic killing by lactoferrin and lysozyme has been reported (André et al., 2015).

Epithelial cells express lysozyme, a major component of granules in neutrophils, which are activated in response to mucosal inflammation (Welsh and Spitznagel, 1971; Cramer and Breton-Gorius, 1987; Cole et al., 2002). Lysozyme has two distinct antibacterial activities: a muramidase activity, which hydrolyzes the bacterial peptidoglycan backbone, leading to degradation of the cell wall and lysis of bacteria; and the non-muramidase portion of lysozyme, which functions as a cationic antimicrobial peptide (Ibrahim et al., 2001; Nash et al., 2006; Davis et al., 2008).

Granulysin is a naturally occurring antimicrobial peptide and is a member of the saposin-like protein (SAPLIP) family that is expressed by activated human natural killer cells and T lymphocytes (Peńa and Krensky, 1997). Recombinant granulysin has shown significant cytotoxicity in both Gram-positive and Gram-negative bacteria (Stenger et al., 1998; Ernst et al., 2000; Wang et al., 2000).

Human defensin 3 possesses broad-spectrum antibacterial activity against many nosocomial pathogens such as *Staphylococcus aureus*, *Enterococcus faecium*, and *Pseudomonas aeruginosa*, as well as clinical isolates of emergent pathogens such as *Stenotrophomonas maltophilia*, *Acinetobacter baumannii*, and drug-resistant microbes (Harder et al., 2001; Hoover et al., 2003; Wu et al., 2003; Maisetta et al., 2006).

Dermcidin is a human-origin antimicrobial peptide that exhibits broad-spectrum activity against *E. coli, E. faecalis, S. aureus*, and *Candida albicans* (Schittek et al., 2001).

Ribonucleases of the T2 family are involved in various biological activities, including scavenging of nucleic acids, degrading self-RNA, serving as extra or intracellular cytotoxins, and modulating host immune responses. However, some RNaseT2 family members lack nuclease activity, suggesting that these proteins have additional functions such as control of the immune system (Luhtala and Parker, 2010). RNase 7 is a novel 14.5-kDa antimicrobial ribonuclease that possesses broad spectrum antimicrobial activity against many pathogenic microorganisms, including vancomycin-resistant *E. faecium* (Harder and Schröder, 2002). Similarly, antimicrobial activity has been reported for human angiogenin (Rnase 5) (Ganz, 2003).

**FIGURE 12:**
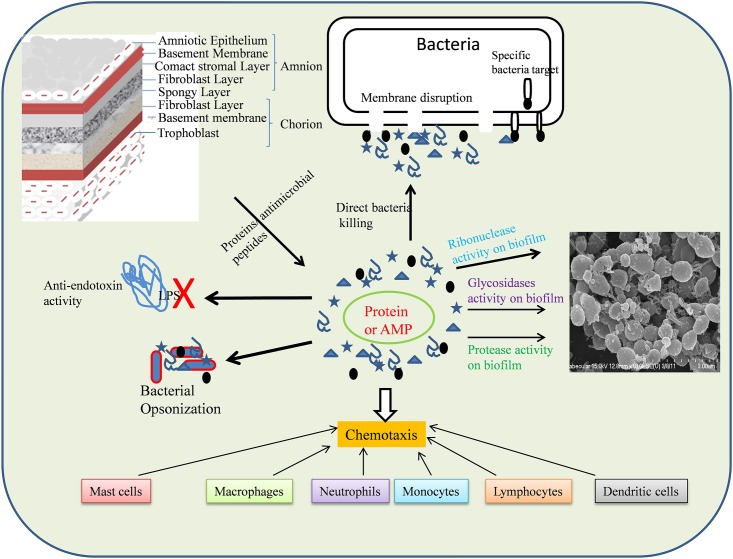
Schematic representation of mode of action AME/CME constituents (Proteins/antimicrobial peptides).

S100A8 and S100A9 are antimicrobial proteins (Raquil et al., 2008) expressed by epithelial cells monocytes, neutrophils, and activated endothelial cells (Barthe et al., 1991; Stríz and Trebichavský, 2004). Heterodimers of these proteins have been suggested to inhibit bacterial adhesion to the mucosal epithelium and bacterial growth through zinc chelation (Santhanagopalan et al., 1995; Nisapakultorn et al., 2001; Lusitani et al., 2003). Altogether, our results indicated that AME/CME possess significant antibacterial and anti-biofilm potential. The antimicrobial effect of AME/CME constituents could be direct or indirect, and could a exert chemotaxic effect on host cells (**Figure 12**).

## Conclusion

The results of this study showed that AME/CME inhibits *S. pneumoniae* growth in a planktonic state and under biofilm conditions. These extracts also possess significant biofilm-eradicating activity. The antimicrobial and antibiofilm activity of AME/CME is a result of several antimicrobial proteins and peptides in the AM/CM. AME/CME overcome the limitation of the whole membranes, in that they are easily preserved and handled could be sterilized through filtration, and could be used together with antibiotics. Although allogeneic tissue has an implicit risk of infectious disease transmission, filter sterilization and screening of the donors for communicable diseases could prevent pathogen transmission.

Amniotic membrane extract and chorionic membrane extract are a natural solution of human origin with no cytotoxicity. Therefore, AME/CME alone or combined with antibiotics could be used to treat *S. pneumoniae* infections such as otitis media.

## Author Contributions

Conceived and designed the experiments: MY, J-JS, S-WC, YG, and SK. Performed the experiments: MY, YG, and SK. Analyzed the data: MY, J-JS, S-WC, and YG. Contributed reagents/materials/analysis tools: S-WC, YG, and SK. Wrote the paper: MY and J-JS.

## Conflict of Interest Statement

The authors declare that the research was conducted in the absence of any commercial or financial relationships that could be construed as a potential conflict of interest.
